# Reduced vascular amyloid burden at microhemorrhage sites in cerebral amyloid angiopathy

**DOI:** 10.1007/s00401-016-1635-0

**Published:** 2016-10-22

**Authors:** Susanne J. van Veluw, Hugo J. Kuijf, Andreas Charidimou, Anand Viswanathan, Geert Jan Biessels, Annemieke J. M. Rozemuller, Matthew P. Frosch, Steven M. Greenberg

**Affiliations:** 1 Department of Neurology, J. Philip Kistler Stroke Research Center, Harvard Medical School, Massachusetts General Hospital, 175 Cambridge Street, Suite 300, Boston, MA 02114 USA; 2 Department of Neurology, Brain Center Rudolf Magnus, University Medical Center Utrecht, Utrecht, The Netherlands; 3 Image Sciences Institute, University Medical Center Utrecht, Utrecht, The Netherlands; 4 Department of Pathology, VU Medical Center, Amsterdam, The Netherlands; 5 Neuropathology Service, C.S. Kubik Laboratory for Neuropathology, Harvard Medical School, Massachusetts General Hospital, Boston, MA USA

**Keywords:** Amyloid β, Microbleeds, Small vessel disease, MRI

## Abstract

Microhemorrhages are strongly associated with advanced cerebral amyloid angiopathy (CAA). Although it has been frequently proposed that the deposition of Aβ in the walls of cortical vessels directly causes microhemorrhages, this has not been studied in great detail, mainly because the ruptured vessels are often missed on routine histopathologic examination. Here, we examined histopathological data from studies targeting microhemorrhages with high-resolution ex vivo 7 T MRI in nine cases with moderate-to-severe CAA, and assessed the presence of Aβ in the walls of involved vessels. We also assessed the density of Aβ positive cortical vessels in areas surrounding microhemorrhages compared to control areas. In seven out of 19 microhemorrhages, the presumed involved vessel could be identified on the histopathological section. Only one of these vessels was positive for Aβ at the site of rupture. Moreover, the density of Aβ positive cortical vessels was lower (1.0 per mm^2^) within a range of 315 µm surrounding the microhemorrhage, compared to control areas (2.0 per mm^2^; *p* < 0.05). These findings question the widely held assumption that the deposition of Aβ in the walls of cortical vessels directly causes microhemorrhages.

**Electronic supplementary material:**

The online version of this article (doi:10.1007/s00401-016-1635-0) contains supplementary material, which is available to authorized users.

## Introduction

Cerebral amyloid angiopathy (CAA) is a common small vessel pathology in the aging human brain. CAA is found in ~30 to 80% of older individuals on autopsy, and has increasingly been recognized as an important contributor to antemortem cognitive decline and dementia [1, 4]. On pathology, CAA is characterized by the deposition of amyloid β (Aβ) in the walls of cerebral leptomeningeal and cortical vessels. Severe CAA is associated with vasculopathic changes, microinfarcts, and microhemorrhages [8, 15, 20, 24]. The latter are considered the hallmark lesions of CAA, especially on MRI. The presence of multiple lobar cerebral ‘microbleeds’ (the MRI signature of old microhemorrhages) as detected on T2*-weighted in vivo MRI is strongly associated with CAA on pathology [17]. Due to the strong association between microhemorrhages and CAA, it is a widely held assumption that the deposition of Aβ in cortical vessels directly causes these vessels to rupture by making them fragile [24]. Accordingly, a longitudinal MRI study in CAA patients showed that new micro- and macrobleeds occurred preferentially at locations with increased levels of Aβ deposition as measured on a previous PiB-PET MRI scan [9]. However, at the neuropathological level, a recent autopsy study did not support a direct topographical relation between microhemorrhages and CAA, after examining histological sections from different brain areas in 113 consecutive autopsy cases [12]. Despite this lack of topographical relation between microhemorrhages and CAA at the microscopic level, it remains unclear if the individual vessels that ruptured and gave rise to those microhemorrhages contained vascular Aβ depositions or not. It has proved to be challenging to assess this, because the ruptured vessels often escape detection on routine pathologic examination.

Here, we used high-resolution ex vivo 7 tesla (T) MRI to ensure successful retrieval of microhemorrhages to study the presence of Aβ in the walls of vessels involved in those microhemorrhages. Moreover, we assessed the density of Aβ positive cortical vessels in areas surrounding microhemorrhages, and compared them to control areas and areas with microinfarcts (another common CAA-related microvascular lesion).

## Methods

### Cases

For this study, we analyzed histopathological data of autopsy cases with CAA previously acquired in the context of two post-mortem MRI studies ([22]; referred to as the Boston data set and [21]; referred to as the Utrecht data set). Both studies were aimed at assessing the histopathology of MRI-observed microbleeds. Inclusion criteria for the Boston data set were presence >10 microbleeds on in vivo clinical MRI and histopathologically confirmed CAA on autopsy (*n* = 5 cases). Inclusion criteria for the Utrecht data set was severe CAA on autopsy (*n* = 5 cases). In both studies, formalin-fixed brain slabs from these cases were subjected to ex vivo 7 T MRI and samples were taken from representative microbleeds for histopathological examination. For the purpose of this study, we selected the samples from cases with moderate-to-severe CAA on pathology only. One case (from the Boston data set) had mild CAA and was, therefore, not considered for the current analysis. Hence, the samples from nine cases were entered into the analysis.

### Ex vivo MRI and sampling

The ex vivo 7 T MRI protocol that was used in both studies included high-resolution T2 and T2*-weighted MRI scans. Representative cerebral microbleeds, identified by one rater using established rating criteria [25], were sampled for histopathological examination. The sampled tissue blocks were processed and embedded in paraffin. Next, 6-μm-thick serial sections were cut on a microtome, guided by MRI. At the level of the microhemorrhage, the first section was stained for hematoxylin and eosin (H&E). Adjacent sections were stained with a monoclonal antibody against Aβ (NAB228 for the Boston data set [13]; anti-Aβ (clone 6F/3D) purchased from DAKO for the Utrecht data set). All retrieved lesions were examined by a neuropathologist (MPF for the Boston data set, AJMR for the Utrecht data set).

### Analysis of the histopathology data

For this study, we selected the histopathological sections containing old or recent microhemorrhages, as confirmed by the neuropathologist on microscopic examination of those sections. As previously described, old microhemorrhages were defined as focal hemosiderin deposits with or without hematoidin, whereas recent microhemorrhages were defined as extravasated intact or (partly) lysed erythrocytes [21, 22].

#### Qualitative assessment of involved vessels

First, we determined if the presumed involved vessel was present on the H&E-stained section, defined as a vessel with evidence of rupture and/or surrounded by erythrocytes or hemosiderin and/or hematoidin deposits (i.e., the microhemorrhage itself). If the involved vessel could be identified, we assessed the presence of Aβ in the wall of that vessel on the adjacent Aβ-stained section.

#### Density of Aβ positive cortical vessels

Second, we assessed the density of Aβ positive cortical vessels in the tissue surrounding the microhemorrhages, see Supplemental Figure 1. A microhemorrhage was excluded for this analysis if another microhemorrhage was present within 3 mm on the same section, to avoid any potential influence. Where possible, for each microhemorrhage, two or three control areas were taken from intact cortical areas harboring CAA but lacking microhemorrhages on the same section (>5 mm away from the microhemorrhage). Photographs were obtained from both the microhemorrhage area, and control areas on the same microscope, through a 1× objective. Additional images with a 2× objective were taken to assist rating. Next, on the obtained images, the areas of interest were defined by manually outlining the cortical ribbon (omitting the underlying white matter and leptomeningeal vessels). Microhemorrhages were masked by manually placing a circular or oval shape covering the microhemorrhage. On the control areas, an identical (in terms of size and shape) ‘placebo’ mask was placed over the cortex in the center of the obtained photograph, thereby avoiding bias for CAA severity in the surrounding areas. One week later, all images were assessed for Aβ positive vessels by one observer (SJvV) blinded to microhemorrhage presence (i.e., the observer did not know whether the circular or oval shape masked a microhemorrhage or not), by placing markers in the center of Aβ positive cortical vessels in an in-house developed interface incorporated in MeVisLab (MeVis Medical Solutions AG, Bremen, Germany). Because the leptomeningeal vessels were not preserved on all sections, these vessels were not analyzed. The area surrounding the masks was divided into six concentric shells each of 315 μm in width (or 50 pixels). The number of Aβ positive cortical vessels per shell was generated by the software. The density of Aβ positive cortical vessels/mm^2^ in each shell was calculated by dividing the number of Aβ positive cortical vessels per shell by the area of that particular shell. Differences between density in microhemorrhage areas and control areas on the same section were assessed by paired samples *t* test per shell, in Graphpad Prism (version 7.00).

## Results

**Fig. 1:**
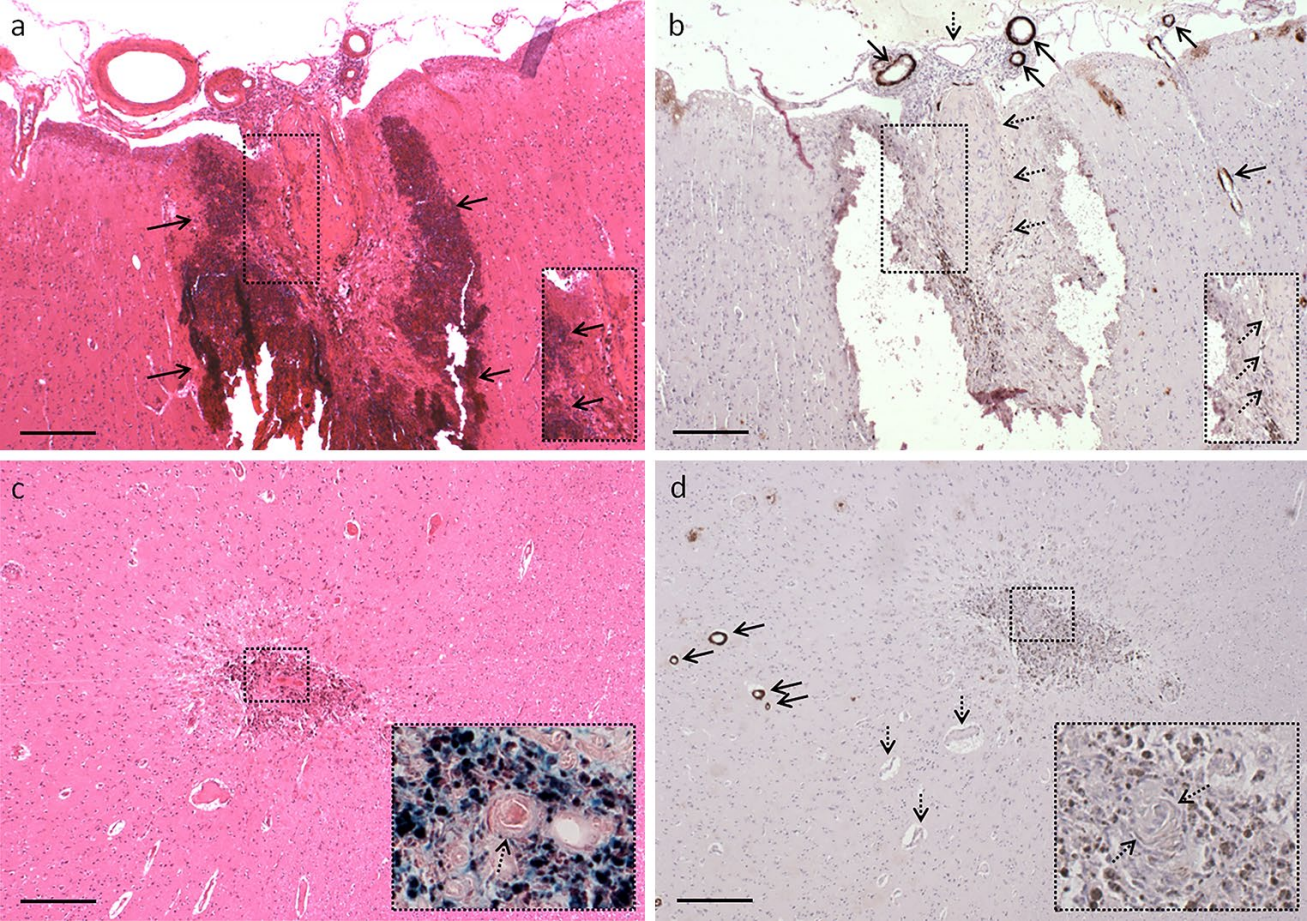
Vascular Aβ was absent in the walls of six out of seven vessels that were involved in microhemorrhages in cerebral amyloid angiopathy. A recent microhemorrhage was identified on an H&E-stained section, characterized by the presence of intact erythrocytes (*arrows*) in the parenchyma, surrounding a penetrating cortical vessel (**a**, *inset* shows enlargement of *boxed area*; adapted from [22] with permission). The adjacent Aβ-stained section revealed that the involved vessel was negative for Aβ (*broken arrows*), whereas neighboring vessels did contain Aβ (*arrows*) (**b**, *inset* shows enlargement of *boxed area*, *broken arrows* indicate absence of Aβ in the wall of this vessel). In another case, an old microhemorrhage was identified on an H&E-stained section, characterized by the focal deposition of hemosiderin (*blue deposits in inset*) in the parenchyma, surrounding a cortical vessel (*broken arrow in inset*) (**c**, *inset* shows Perl’s Prussian *blue* staining of the area outlined in the *box* for enhanced detection of hemosiderin). The adjacent Aβ-stained section revealed that the involved vessel was negative for Aβ (*broken arrows in inset*), as well as vessels in close proximity to the microhemorrhage (*broken arrows*), whereas vessels further away did contain Aβ (*arrows*) (**d**, *inset* shows enlargement of *boxed area*). All *scale bars* indicate 250 µm

**Fig. 2:**
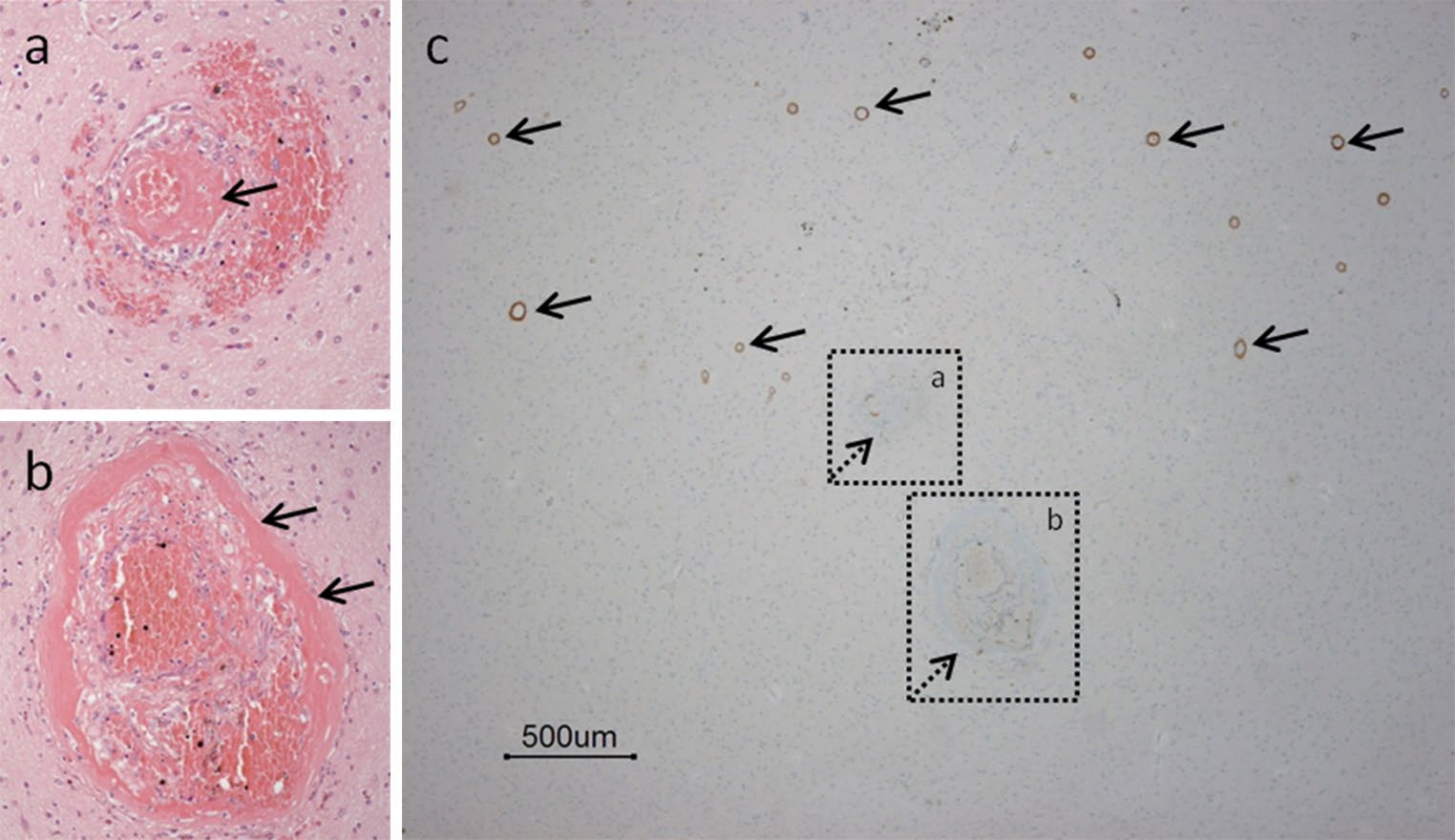
Vascular Aβ was absent in the walls of vessels with fibrinoid necrosis in cerebral amyloid angiopathy. Two severely dilated vessels with fibrinoid necrosis (*arrows*, also confirmed by phosphotungstic acid/hematoxylin stain, not shown) were identified on H&E (**a**, **b**) (**b** was adapted from [21] with permission). The adjacent Aβ-stained section revealed that these vessels were negative for Aβ (*broken arrows*), whereas neighboring vessels did contain Aβ (**c**). *Boxed areas* in **c** represent areas contained in **a** and **b** respectively

In total, 19 ex vivo MRI-targeted microhemorrhages were available for analysis (nine from the Boston data set and ten from the Utrecht data set). On histopathology, 12 proved to be old, and seven acute microhemorrhages. In seven (three acute and four old) out of 19 microhemorrhages, the presumed involved vessel could be identified on the adjacent Aβ-stained section. In six out of these seven vessels, the walls were negative for Aβ (Fig. 1). Interestingly, in one autopsy case, four additional vessels with fibrinoid necrosis (confirmed by phosphotungstic acid/hematoxylin stain) were observed, for which adjacent Aβ-stained sections were available. None of these vessels were positive for Aβ (Fig. 2).

**Fig. 3:**
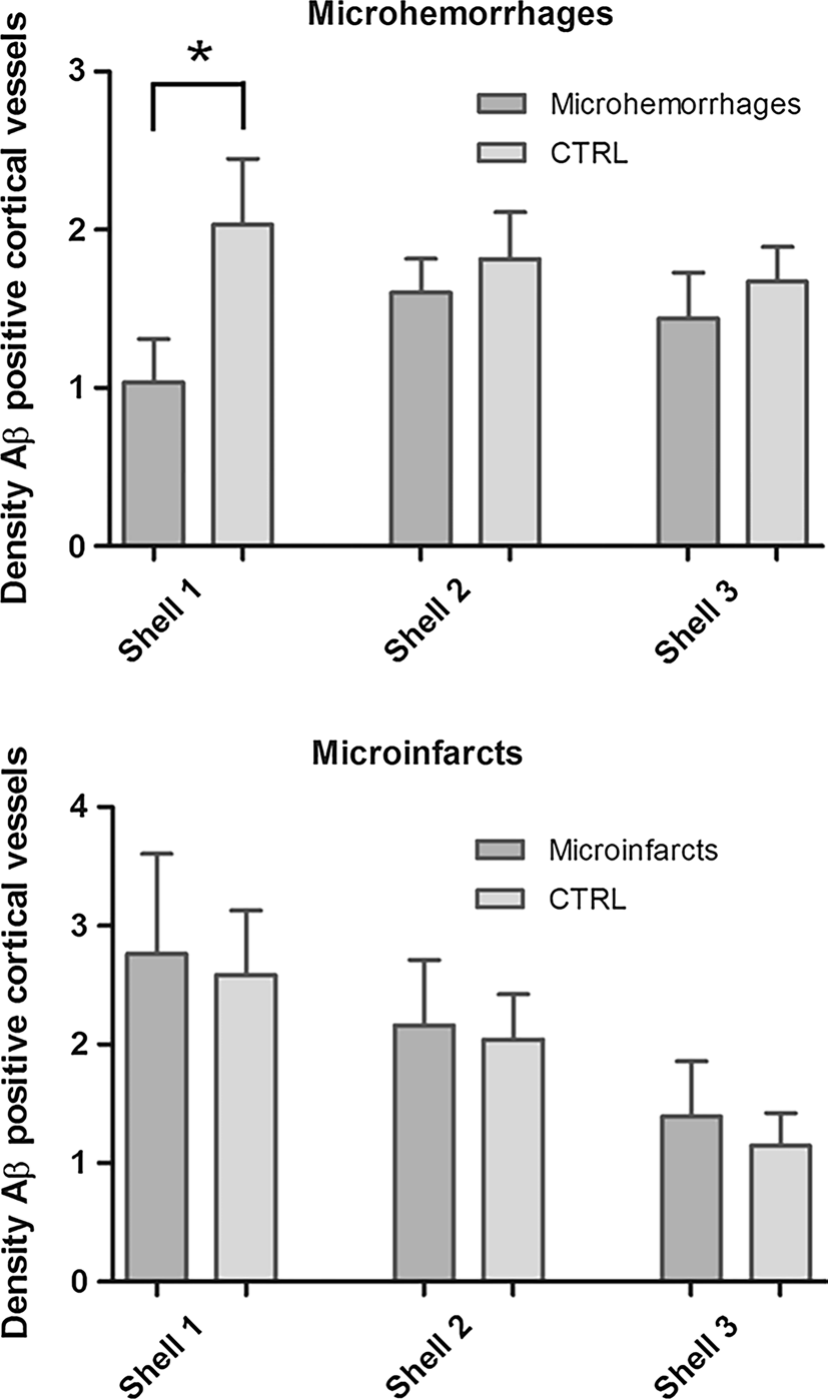
Density (vessels/mm^2^) of Aβ positive cortical vessels was lower in the shell directly adjacent to a microhemorrhage, compared to shells further away. The first shell adjacent to a microhemorrhage contained a lower density of Aβ positive cortical vessels compared to control areas (*upper graph*). This effect was not observed in shells further away from the lesion. Moreover, the first shell adjacent to a microinfarct did not contain a lower density of Aβ positive cortical vessels compared to control areas (*lower graph*). This suggests that the observed lower density of Aβ positive cortical vessels adjacent to a microhemorrhage was not caused by tissue damage in that shell. *p < 0.05 (paired samples *t* test). *Error bars* represent SEM. The graphs depict the first three shells. The width of one shell was set at 50 pixels, which equals 315 μm. Microhemorrhages: *n* = 17; total number of CTRL areas: *n* = 34; microinfarcts: *n* = 8

Next, we hypothesized based on the initial qualitative results that microhemorrhages might occur preferentially in CAA-sparse microareas. Hence, we assessed the density of Aβ positive cortical vessels in shells close to microhemorrhages compared to shells further away. Two microhemorrhages were excluded, because they were located in close proximity (<3 mm) to another microhemorrhage, leaving 17 microhemorrhages for analysis. Per microhemorrhage area, at least one or two control areas were chosen from the same histopathological section. If ≥2 control areas were taken on one section, the average densities (per shell) for those control areas were entered into the paired samples analysis. Only the shell directly adjacent (<315 μm) to a microhemorrhage had a lower density of Aβ positive cortical vessels compared to its control shell (*t*(16) = 2.152; *p* < 0.05) (Fig. 3). For shells further away from the microhemorrhage, we found no difference in density from controls. We repeated the analysis after standardizing the density for each area, to see if this changed the results. First, we calculated the average density of all six shells. Next, we divided the density of Aβ positive cortical vessels for each shell, by the average density of all six shells. This approach did not change the results.

Finally, we assessed whether the observed lower density of Aβ positive cortical vessels could be explained by local tissue damage as a result of the microhemorrhage. Hence, we repeated the analysis around microinfarcts (*n* = 8), another frequently observed CAA-related microvascular lesion causing local tissue damage (Supplemental Fig. 2). We found no difference between microinfarct areas and control areas (Fig. 3). This suggests that the observed lower density of Aβ positive cortical vessels close to a microhemorrhage is not likely due to tissue damage caused by the lesion in that area.

## Discussion

In this exploratory observational histopathological study, we found that only one out of seven preserved vessels that were involved in microhemorrhages contained Aβ. Moreover, we observed a lower density of Aβ positive cortical vessels in the microenvironment closest to the microhemorrhages, suggesting that microhemorrhage formation may not be a direct consequence of more severe CAA locally, and indeed may occur preferentially in areas of relatively low CAA. These findings question a frequently proposed mechanism that microhemorrhage formation in CAA is a direct consequence of Aβ deposition in the walls of the responsible vessels.

There is no doubt that hemorrhages are manifestations strongly associated with CAA, as supported by a large body of the literature (e.g., [11, 23, 24]). Nevertheless, it remains unclear what exact mechanisms drive a particular vessel segment in a brain with advanced CAA to bleed. At the larger spatial scale resolved by MRI and amyloid-PET (>2 mm^2^), higher levels of amyloid are spatially associated with MRI-observed microbleeds [3, 9], but only a few studies have directly investigated this relation at the single vessel level. A recent neuropathology study that assessed the relation between microhemorrhages and CAA in sections obtained at autopsy for routine neuropathologic examination found no significant association between the presence of CAA and microhemorrhages in any brain region that was examined [12]. Although the authors did not investigate the vessels directly involved in those microhemorrhages, they noted that microhemorrhages were mainly observed at the cortical-white matter junction, whereas CAA was restricted to superficial and short penetrating cortical vessels.

Capturing a microhemorrhage, and in particular, the involved vessel of a microhemorrhage on routine pathological examination of the autopsied brain in CAA is difficult. Microhemorrhages are not frequently captured in routine sections, and therefore often missed upon neuropathological examination. Moreover, the majority of microhemorrhages that are captured in a section are more likely to be old and (partly) degraded. For those reasons, there are only a few examples where the presence of ruptured vessels on a section in the context of CAA is mentioned. Probably, one of the first reports was by Vonsattel and colleagues [24]. In this paper, 136 brains without hemorrhage and 17 brains with CAA with hemorrhage were examined. Interestingly, the authors report findings from serial sections taken from one case with CAA and multiple microhemorrhages. These serial sections disclosed a direct relationship between fibrinoid necrosis and the site of rupture of the vessel wall, with or without erythrocyte extravasation. The authors noted that these vessels were congophilic, but it remains unclear if the vascular Aβ deposits were observed at the site of rupture or more remotely. More recently, a postmortem MRI study targeting MRI-observed microbleeds for histopathological analysis in CAA, mentioned that for several corresponding histopathologically confirmed hemorrhages, it was possible to determine which vessel ruptured. Aβ staining in those vessels demonstrated the presence of Aβ deposits in the walls of involved vessels [19]. Others, however, did not observe Aβ at sites of rupture [7, 16]. One study analyzed vessels in 3D after serial sectioning and found that at the site of rupture, the vessels were dilated and exhibited fibrinoid necrosis in the absence of vascular Aβ [10, 16]. Interestingly, the vessels did contain Aβ further upstream and downstream in the same vessel.

To increase retrieval of ruptured vessels, we made use of high-resolution ex vivo MRI to target lesions for detailed histopathological analysis. Moreover, we performed serial sectioning to capture the site of rupture on a single thin section [5]. Even using this targeted approach, 12 out of 19 ruptured vessels were still unavailable for detailed histopathologic examination, either because they were lost in the process of sectioning or because they had deteriorated over time, which is more likely in the case of old microhemorrhages. Hence, it cannot be ruled out that these vessels did contain Aβ before they ruptured. However, the fact that only one out of seven available involved vessels was positive for Aβ suggests that it is unlikely that Aβ always directly causes vessels to rupture and calls for different additional explanatory mechanisms by which CAA indirectly results in microhemorrhage formation.

One possible explanation for our observations is that the extravasation of erythrocytes pushes away the Aβ deposits from the vessel wall, hence rendering the site of rupture amyloid-negative on subsequent histopathological examination. Another possible explanation could be that early immune reactions, such as the infiltration of macrophages, result in clearing of Aβ in the area. However, the absence of Aβ in the walls of recent microhemorrhages as well as yet unruptured vessels with fibrinoid necrosis, a pathologic change that is strongly associated with microhemorrhage formation [24], does not support these explanations. Moreover, the observation that the density of Aβ positive cortical vessels was lower directly adjacent to a microhemorrhage also supports that the lesion was not caused by more severe CAA locally. Finally, the fact that we did not observe a similar lower density of Aβ positive cortical vessels close to microinfarcts suggests that this was unlikely due to tissue damage in the adjacent area of the microvascular lesion. A more likely explanation for our findings would be that vascular Aβ deposition does not cause vessels to rupture directly or locally, but that it may have an indirect effect on the integrity of the microvascular network. A known consequence of CAA is impaired autoregulation (e.g., dysfunctional regulation of cerebral blood flow), which may lead to weak spots in the microvasculature in the case of alterations in systemic blood pressure [2, 6]. It is conceivable that such blood pressure changes increase the risk of rupture at weak vessel segments without vascular Aβ deposits. If this is true, one would expect to find vascular Aβ deposits in vessels upstream or downstream of the site of rupture. This would also explain previous observations of Aβ positive vessels associated with hemorrhages [19, 24] and spatial associations between amyloid-PET imaging and MRI-observed microbleeds [3, 9].

This study has several limitations. First, we cannot rule out that there may have been some bias in selecting the control areas, unconsciously in CAA-rich regions. Second, the presence of Aβ in the walls of vessels was assessed on a single thin section stained for Aβ. Hence, no information was available on the segments of the same vessels above and below those sections. Future studies are warranted to verify our observations in larger volumes of tissue, by analyzing the cortical microvascular network in 3D. This can for instance be achieved by optically clearing samples, as recently demonstrated for formalin-fixed human tissue [18], as well as for a whole mouse brain [14]. Interestingly, the latter study reported small foci of hemosiderin deposits in close proximity to vessels with CAA by means of 3D reconstructions of individual vessels in a mouse model of Alzheimer's disease [14]. Third, because this study consisted of a relatively small data set, there is a possibility of a false-positive finding. However, the shell-analysis was performed with a pre-specified hypothesis and provided independent confirmation of the initial independent qualitative finding of absent CAA at bleeding sites. Finally, it could be possible that the microhemorrhages observed in our study were the result of other (age-related) pathological processes that often coexist with CAA, such as hypertensive arteriopathy. Other factors that might have influenced some of our observations are prolonged formalin exposure as well as secondary effects of therapeutic interventions prior to death. Moreover, extending our findings in the context of other (hereditary) small vessel diseases with associated microhemorrhages would be an interesting topic for further research.

## Conclusions

Our findings question a widely held assumption that the deposition of Aβ in the walls of cortical vessels directly causes microhemorrhages. Further studies are needed to validate this observation in larger data sets and to determine the exact mechanisms by which vascular Aβ leads to hemorrhage formation in CAA.

## Electronic supplementary material

Below is the link to the electronic supplementary material.

Supplementary material 1 (DOCX 3729 kb)

## References

[1] Arvanitakis Z, Leurgans SE, Wang Z, Wilson RS, Bennett DA, Schneider JA (2011). Cerebral amyloid angiopathy pathology and cognitive domains in older persons. Ann Neurol.

[2] Blinder P, Tsai PS, Kaufhold JP, Knutsen PM, Suhl H, Kleinfeld D (2013). The cortical angiome: an interconnected vascular network with noncolumnar patterns of blood flow. Nat Neurosci.

[3] Dierksen GA, Skehan ME, Khan MA (2010). Spatial relation between microbleeds and amyloid deposits in amyloid angiopathy. Ann Neurol.

[4] Ellis RJ, Olichney JM, Thal LJ (1996). Cerebral amyloid angiopathy in the brains of patients with Alzheimer's disease: the CERAD experience, part XV. Neurology.

[5] Fisher CM (2003). Hypertensive cerebral hemorrhage. Demonstration of the source of bleeding. J Neuropathol Exp Neurol.

[6] Fisher M (2016). Cerebral microbleeds and thrombolysis: clinical consequences and mechanistic implications. JAMA Neurol.

[7] Fisher M, French S, Ji P, Kim RC (2010). Cerebral microbleeds in the elderly: a pathological analysis. Stroke.

[8] Greenberg SM, Al-Shahi Salman R, Biessels GJ (2014). Outcome markers for clinical trials in cerebral amyloid angiopathy. Lancet Neurol.

[9] Gurol ME, Dierksen G, Betensky R (2012). Predicting sites of new hemorrhage with amyloid imaging in cerebral amyloid angiopathy. Neurology.

[10] Itoh Y, Yamada M (1997). Cerebral amyloid angiopathy in the elderly: the clinicopathological features, pathogenesis, and risk factors. J Med Dent Sci.

[11] Knudsen KA, Rosand J, Karluk D, Greenberg SM (2001). Clinical diagnosis of cerebral amyloid angiopathy: validation of the Boston criteria. Neurology.

[12] Kövari E, Charidimou A, Herrmann FR (2015). No neuropathological evidence for a direct topographical relation between microbleeds and cerebral amyloid angiopathy. Acta Neuropathol Commun.

[13] Lee EB, Leng LZ, Zhang B (2006). Targeting amyloid-beta peptide (Abeta) oligomers by passive immunization with a conformation-selective monoclonal antibody improves learning and memory in Abeta precursor protein (APP) transgenic mice. J Biol Chem.

[14] Lo P, Crouzet C, Vasilevko V, Choi B (2016). Visualization of microbleeds with optical histology in mouse model of cerebral amyloid angiopathy. Microvasc Res.

[15] Love S, Chalmers K, Ince P (2014). Development, appraisal, validation and implementation of a consensus protocol for the assessment of cerebral amyloid angiopathy in post-mortem brain tissue. Am J Neurodegener Dis.

[16] Maeda A, Yamada M, Itoh Y, Otomo E, Hayakawa M, Miyatake T (1993). Computer-assisted three-dimensional image analysis of cerebral amyloid angiopathy. Stroke.

[17] Martinez-Ramirez S, Romero J-R, Shoamanesh A (2015). Diagnostic value of lobar microbleeds in individuals without intracerebral hemorrhage. Alzheimer's Dement.

[18] Murray E, Cho JH, Goodwin D (2015). Simple, scalable proteomic imaging for high-dimensional profiling of intact systems. Cell.

[19] Schrag M, McAuley G, Pomakian J (2010). Correlation of hypointensities in susceptibility-weighted images to tissue histology in dementia patients with cerebral amyloid angiopathy: a postmortem MRI study. Acta Neuropathol.

[20] Soontornniomkij V, Lynch MD, Mermash S (2004). Cerebral microinfarcts associated with severe cerebral beta-amyloid angiopathy. Brain Pathol.

[21] Van Veluw SJ, Biessels GJ, Klijn CJ, Rozemuller AJ (2016). Heterogeneous histopathology of cortical microbleeds in cerebral amyloid angiopathy. Neurology.

[22] Van Veluw SJ, Charidimou A, Van der Kouwe AJ (2016). Microbleed and microinfarct detection in amyloid angiopathy: high-resolution MRI-histopathology study. Brain.

[23] Viswanathan A, Greenberg SM (2011). Cerebral amyloid angiopathy in the elderly. Ann Neurol.

[24] Vonsattel JP, Myers RH, Hedley-Whyte ET (1991). Cerebral amyloid angiopathy without and with cerebral hemorrhages: a comparative histological study. Ann Neurol.

[25] Wardlaw JM, Smith EE, Biessels GJ (2013). Neuroimaging standards for research into small vessel disease and its contribution to ageing and neurodegeneration. Lancet Neurol.

