# Supplementary material for: Reduced vascular amyloid burden at microhemorrhage sites in cerebral amyloid angiopathy
Source: Acta Neuropathol. 2016 Oct 22;133(3):409–15. doi: 10.1007/s00401-016-1635-0 (PMC5325834; doi:10.1007/s00401-016-1635-0)
Supplement: Supplementary file 1 — Supplementary material 1 (DOCX 3729 kb) [file 401_2016_1635_MOESM1_ESM.docx]

**Title: Reduced vascular amyloid burden at microhemorrhage sites in cerebral amyloid angiopathy**

**Journal: Acta Neuropathologica**

Susanne J. van Veluw PhD^1,2^, Hugo J. Kuijf PhD^3^, Andreas Charidimou MD PhD^1^, Anand Viswanathan MD PhD^1^, Geert Jan Biessels MD PhD^2^, Annemieke J.M. Rozemuller MD PhD^4^, Matthew P. Frosch MD PhD^5^, Steven M. Greenberg MD PhD^1^

*^1^ J. Philip Kistler Stroke Research Center, Department of Neurology, Massachusetts General Hospital and Harvard Medical School, Boston, MA, USA*

*^2^ Department of Neurology, Brain Center Rudolf Magnus, University Medical Center Utrecht, Utrecht, the Netherlands*

*^3^ Image Sciences Institute, University Medical Center Utrecht, Utrecht, the Netherlands*

*^4^ Department of Pathology, VU Medical Center, Amsterdam, the Netherlands*

*^5^ Neuropathology Service, C.S. Kubik Laboratory for Neuropathology, Massachusetts General Hospital and Harvard Medical School, Boston, MA, USA*

Corresponding author:

Susanne J. van Veluw, svanveluw@mgh.harvard.edu

**
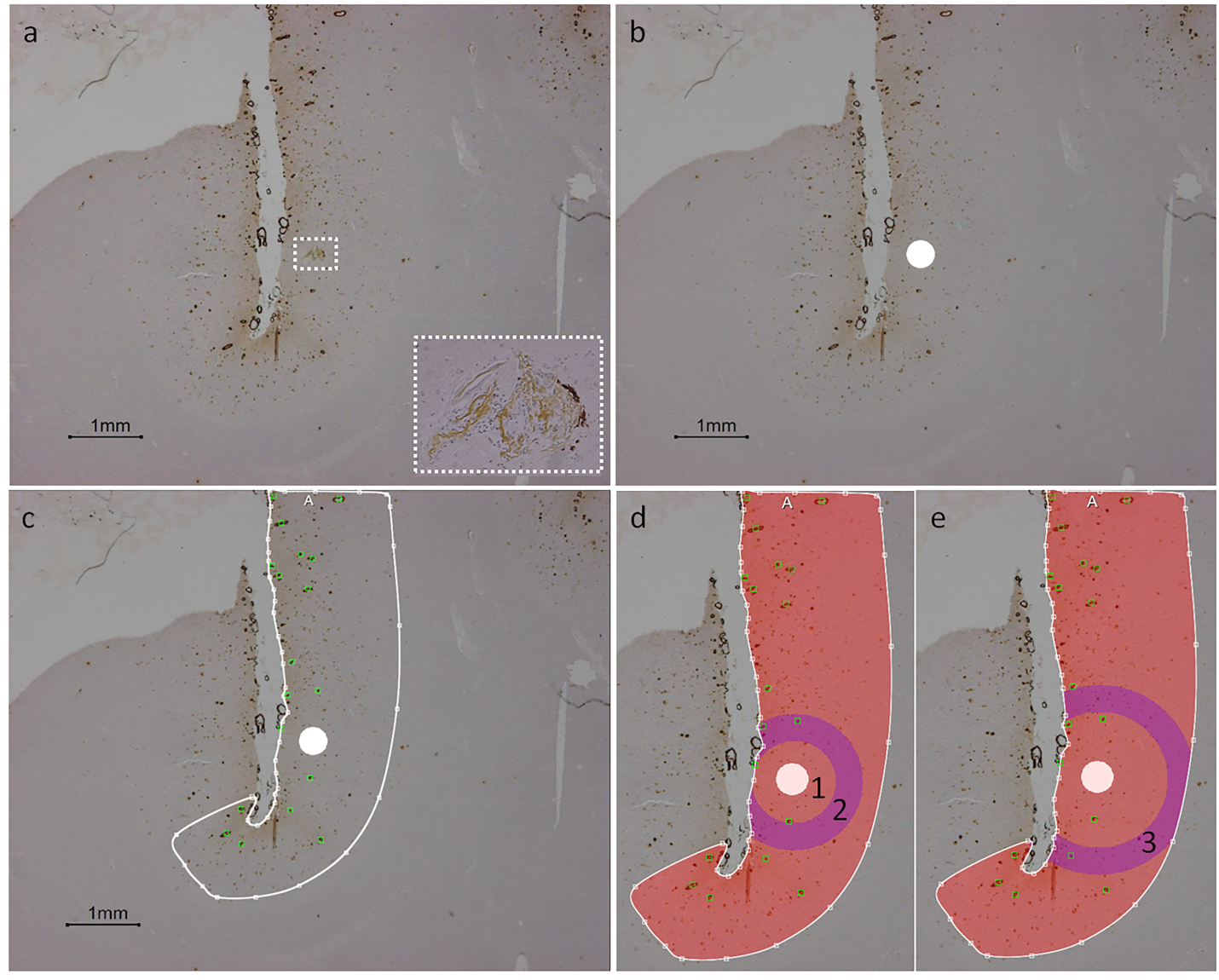
**

**Supplemental Figure 1.** Example of the analysis pipeline for one area containing a microhemorrhage.

An old microhemorrhage (a; inset shows enlargement of boxed area) was manually covered by a mask (b). Next, the cortical ribbon was manually outlined and one rater placed markers (in green) on the center of each Aβ positive cortical vessel in that region (c). Next, the software provided number of markers within each shell surrounding the mask (d, e). In total, six shells were generated, each measuring 50 pixels (~315 µm) in width. Here, the first three shells are indicated. Density of Aβ positive cortical vessels per shell: markers / (sum of pixels · pixel size).

Results for this area:

| Shell | Markers | Sum of pixels | Density *(1 pixel = 0.0063 · 0.0063 mm^2^)* |
| --- | --- | --- | --- |
| 1-50 | 1 | 16096 | 1.5509 |
| 50-100 | 2 | 24499 | 2.0380 |
| 100-150 | 1 | 28009 | 0.8913 |
| 150-200 | 3 | 26882 | 2.7860 |
| 200-250 | 2 | 28337 | 1.7619 |
| 250-300 | 0 | 28207 | 0 |
| **TOTAL** | **9** | **152093** | - |

Average density in this area: 9 / (152093 · 1 pixel) = 1.4772 vessel / mm^2^


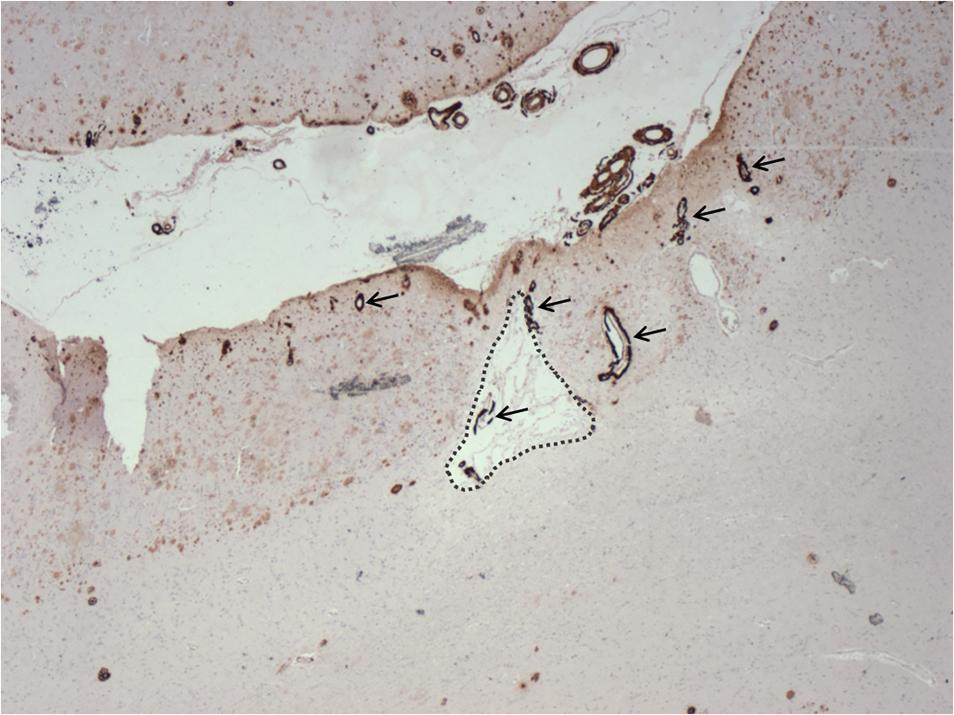


**Supplemental Figure 2.** Microinfarct.

A chronic cortical microinfarct (outlined in grey dotted line) is shown on an Aβ-stained section. The vessels in close proximity to the microinfarct are positive for Aβ (arrows).
